# Reproductive Biology Including Evidence for Superfetation in the European Badger *Meles meles* (Carnivora: Mustelidae)

**DOI:** 10.1371/journal.pone.0138093

**Published:** 2015-10-14

**Authors:** Leigh A. L. Corner, Lynsey J. Stuart, David J. Kelly, Nicola M. Marples

**Affiliations:** 1 School of Veterinary Medicine, University College Dublin, Dublin, Ireland; 2 Department of Zoology, School of Natural Sciences, Trinity College Dublin, Dublin, Ireland; 3 Trinity Centre for Biodiversity Research, Trinity College Dublin, Dublin, Ireland; University of Jyväskylä, FINLAND

## Abstract

The reproductive biology of the European badger (*Meles meles*) is of wide interest because it is one of the few mammal species that show delayed implantation and one of only five which are suggested to show superfetation as a reproductive strategy. This study aimed to describe the reproductive biology of female Irish badgers with a view to increasing our understanding of the process of delayed implantation and superfetation. We carried out a detailed histological examination of the reproductive tract of 264 female badgers taken from sites across 20 of the 26 counties in the Republic of Ireland. The key results show evidence of multiple blastocysts at different stages of development present simultaneously in the same female, supporting the view that superfetation is relatively common in this population of badgers. In addition we present strong evidence that the breeding rate in Irish badgers is limited by failure to conceive, rather than failure at any other stages of the breeding cycle. We show few effects of age on breeding success, suggesting no breeding suppression by adult females in this population. The study sheds new light on this unusual breeding strategy of delayed implantation and superfetation, and highlights a number of significant differences between the reproductive biology of female Irish badgers and those of Great Britain and Swedish populations.

## Introduction

The reproductive biology of the European badger (*Meles meles* Linnaeus) is of particular interest because it is one of the 2% of mammalian species that show delayed implantation [1, 2]. Delayed implantation is important because it allows the disassociation of timing of conception from the subsequent births. Superfetation, which is the finding of young at different stages of development within the uterus [3], or superfetation-like conditions have been reported for numerous species including humans, domestic livestock, rodents and mustelids [3]. However the badger has been suggested as one of only five eutherian species which shows natural superfetation as a reproductive strategy, the others being American mink (*Neovison vison* Schreber,), casiragua (*Proechimys semispinosus* Tome), North African gundi (*Ctenodactyles gundi* Rothmann), brown hare (*Lepus europaeus* Pallas), and possibly the common tenrec (*Tenrec ecaudatus* Schreber) [3]. Superfetation after delayed implantation is a very unusual reproductive system but is of great importance for understanding the selective pressures on both the male and female mating tactics, since it would allow females to mate multiple times and facilitate fertilisation by more than one male, allowing females to practice cryptic polyandry [4]. This deceptive type of polyandry is likely to reduce the risk of infanticide by males as they would be uncertain of their paternity of any offspring produced. The badger is of economic as well as ecological importance since they are a reservoir of tuberculosis (*Mycobacterium bovis* Karlson & Lessel) infection [5, 6]. Studies of reproduction in Irish badgers is of particular interest because this medium density population has not received much attention despite the fact they show interesting genetic and morphological differences from British populations, where the suggested cases of superfetation have previously been identified [4].

The European badger is found in highly diverse habitats across Europe [7]. Population densities vary from abundant in some areas of Britain (up to 44.3 individuals / km^2^ [8], through medium densities in Sweden (2.8 individuals / km^2^ [9] to low in some areas of Continental Europe (Russia 0.46 individuals / km^2^ [10]; Spain 0.28 individuals /km^2^ [11]; Poland 0.16 individuals /km^2^ [12]). The Irish badger population is of medium density (1.9 individuals/km^2^ [13]) consisting of small social groups comprising between three and four individuals [14]. The social groups hold contiguous territories, but the level of territoriality is low and incursions between groups are frequent [15, 16].

The reproductive biology of the European badger has been described for many parts of their range [17–21], including Ireland [22]. These studies on female badgers have used visual examination of the reproductive tract and histology to describe the reproductive cycle. Reproduction has been described in low [18], medium [22] and high density populations [19]. Badgers are polyoestrous, having multiple oestrous cycles throughout the year [4]. The female badger has developed a reproductive strategy whereby fertilization is not immediately followed by implantation but by suspended, very slow or delayed embryogenesis in the blastocyst (‘embryonic diapause’; [1, 4, 23, 24]) which may extend for up to 11 months. Such ‘delayed implantation’ is rare amongst mammals, but common within the mustelid family [4, 23–25]. In the European badger, the blastocysts remain free within the uterine lumen and implantation occurs over a restricted period from December to February, varying with the geographic area [4, 26–28]. Implantation is triggered by photoperiod as demonstrated experimentally by Canivenc and Bonnin [29]. The duration of gestation, that is, the period from fertilization to birth, is variable depending on the time of ovulation and the time of implantation. Delayed implantation allows births and post-natal development of cubs to occur at a time of the year favourable to their development [30]. The duration of post-implantation foetal development is short (47–51 days), and cubs are weaned during spring, a period of nutritional abundance [30].

Further ovulations after the first blastocyst has been formed, and further matings afford an opportunity for superfetation to occur. Superfetation takes place when additional conceptions occur during an existing pregnancy [3]. This leads to the existence of embryos of different developmental stages within the female reproductive tract and the embryos may have different fathers. This may be of benefit to females in two ways. It may allow them to practice cryptic mate choice, mating with multiple males but only using some of those matings for fertilisation, and it would also raise the uncertainty of paternity among the resident males in a group, so reducing the risk of infanticide [4]. A better understanding of superfetation with embryonic diapause, may help to explain the mating behaviours and territoriality of both male and female badgers [4].

Although Irish badgers may be genetically distinct from badgers in the UK [31], are morphologically different [32], and differ markedly in their diet [33], previous interpretation of the evidence suggests no reproductive differences between the two populations [22]. The present study aimed to investigate in greater detail than ever before whether the reproductive biology of female Irish badgers was significantly different from that found in other populations, and to look for evidence of superfetation.

## Materials and Methods

### Badgers

The badgers were captured between March 2005 and September 2006. The badgers for examination were selected randomly from those available after badgers with visible lesions of tuberculosis were excluded. Badgers originated from 20 of the 26 counties in the Republic of Ireland, with the majority taken from counties Donegal, Cavan, Monaghan, and Cork and Clare. The badgers in the study were killed during the culling programme undertaken by the Department of Agriculture, Food and the Marine (DAFM) as part of the national cattle tuberculosis control program [34]; none were killed for the purposes of this study. The badgers were killed by a trained DAFM marksmen under a series of licences issued by the National Parks and Wildlife Service to DAFM (Licence numbers 11a 2005, 11a2006, 14DC2005, 14DC 2006, 33VN2005 and 33VN2006).

Tooth wear was used to estimate age. Using the degree of wear and discolouration of the incisors, canines, premolars and molars on both mandibles [35], individuals were classified as juvenile (<1 year), yearling (1–2 years), adult (2–4 years) or aged adults (> 4 years).

### Post Mortem Examination

At the post mortem examination the badgers were weighed and the complete reproductive tract was removed. Each horn of the uterus was flushed with 10ml of normal saline to remove blastocysts. The flushings were collected in a petri dish and examined for blastocysts using a low power (ranging from 6.3 to 57x magnification as needed) stereoscopic binocular microscope. The diameter of each blastocyst was measured (mm) using a low power biocular microscope and graticule. The full length of each uterine horn was incised and the internal surface examined by eye for retained blastocysts, however all blastocysts were washed out with the original flushing procedure. We are confident that there were no retained blastocysts as even the smallest ones found in the flushings would have been readily visible. In cases when females were gravid, the number of foetuses was counted. Sows were grouped into one of four mutually exclusive reproductive states: those with blastocysts, those that were gravid (that is, foetuses present), those post parturient (involuting uterus, increased length of horn, thickened walls and prominent black placental scars) and those not pregnant (no blastocysts and non-gravid). A post mortem blood sample was collected from the heart and serum was extracted by centrifugation.

The ovaries, uterus, and vagina were fixed in 10% buffered formalin for histology. After fixation the ovaries were cut longitudinally into three 1mm thick sections. The sections were processed for histology along with a transverse section of each of the other organs. The fixed tissues were embedded in paraffin wax and sections were stained with haematoxylin and eosin.

### Histological Examination

Females used in this study were determined to be in oestrus based on the cellular composition of the endometrium and the vaginal epithelium [36, 37]. Criteria used for recognising oestrus in the endometrium were a thickened endometrium with a simple columnar luminal epithelium, highly developed glands that were fully enlarged and located at the interior edge and periphery of the endometrium. The criteria for recognising oestrus in the vaginal epithelium were a very thick (>3 layers), stratified squamous epithelium, keratinised cells at the surface which were visible in the vaginal lumen as exfoliated cells [36, 37].

Sections of the ovaries were examined for secondary and tertiary follicles and for corpora lutea [37, 38]. The total numbers of each of these structures in the ovaries was estimated.

### Serum progesterone

Serum progesterone concentrations were determined using a time-resolved fluorescent immunoassay (FIA) using an AutoDELFIA Progesterone kit (Perkin Elmer, Wallac Oy, Turku, Finland), as described previously [39]. All samples were assayed across four plates within a single assay with a sensitivity of 0.01 ng progesterone /mL. A serial dilution of a high badger serum progesterone sample with assay diluent showed parallel measurements to the standard curve. The mean intra-plate coefficients of variation (CV) were 2.89% (high), 1.46% (medium) and 7.36% (low), for quality control (QC) sera pools containing 2.02 ng progesterone/ml (High), 1.54 ng progesterone/ml (Medium) and 0.38 ng progesterone/ml (Low), respectively. The inter-plate CV for the same QC pools were 4.08%, 4.05% and 8.96%, respectively.

### Statistical Analysis

For descriptive and analytical purposes, the first month of the breeding year was deemed to be March. All analyses were performed in R [40]. We selected non-parametric tests for two-sample comparisons, as we were generally testing small datasets (n < 100) [41]. Where 2-way tests were required, we relied on 2-way ANOVA, as it has been shown to be more reliable [42] and more conservative [43] than the non-parametric alternative [44]. A binomial regression on the presence of corpora lutea was conducted with the glm function in R (family = binomial).Figures were plotted in R or GraphPad Prism version 4 (San Diego California USA, www.graphpad.com).

## Results

### Badgers

During the study period, of the 1489 badgers available, the reproductive tract of 264 female badgers randomly chosen from each month of the year were examined: six were juveniles, 96 yearlings, 97 adult and 65 aged adults (Table 1). Body weights varied during the course of the year, being highest in late autumn (November, mean weight 11.63kg) and lowest in early summer (June, mean weight 7.46kg). Across both years of the study the badgers were significantly heavier in winter (Dec, Jan, Feb; n = 101) than in summer (Jun, Jul, Aug; n = 42) (Wilcoxon rank sum test with continuity correction W = 524, N = 97, 41, p <0.001). Cubs were excluded from this analysis as they only reach adult bodyweights by the end of their first year (Lueps, 1983).

**Table 1:** Number of female badgers examined each calendar month between March 2005 and September 2006.

Age	Month												Total
	Jan	Feb	Mar	Apr	May	June	July	Aug	Sept	Oct	Nov	Dec	
Juvenile	3	1	0	0	0	1	1	0	0	0	0	0	6
Yearling	12	20	7	8	7	6	5	5	8	11	1	6	96
Adult	13	16	15	9	6	4	6	4	5	9	2	8	97
Aged	5	8	14	5	1	4	4	4	1	5	5	9	65
Total	33	45	36	22	14	15	16	13	14	25	8	23	264

Females were defined as pregnant if they carried blastocysts or foetuses (that is, were gravid). Juveniles showed ovarian activity that was restricted to the presence of secondary and tertiary follicles, but no corpora lutea and they had an immature uterus. With the exception of the non-pregnant state, the reproductive status of yearlings, adults and aged adult sows was restricted to specific times of the year: those with blastocysts (March—December), those that were gravid (January—February) and those postpartum (January—February). Non-pregnant sows (no blastocysts and non-gravid) were found throughout the year.

### Reproductive pattern

**Fig 1:**
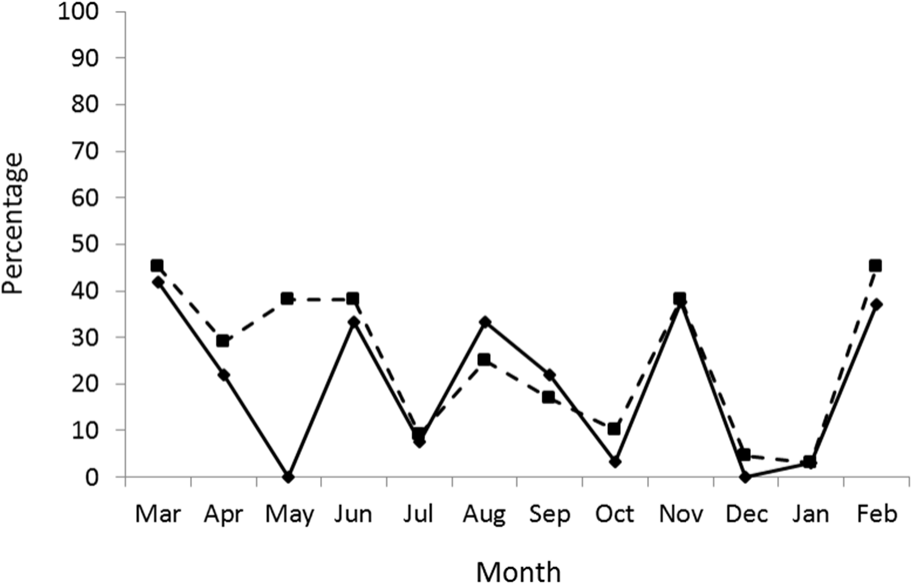
The proportion of females by month in oestrus. The proportion of females in each month in oestrus, as determined by histological examination of the endometrium (solid line) and the vaginal epithelium (broken line).

Sows in oestrus were seen throughout the year but the proportion in each month varied widely, from 0 to 42%, with no clear seasonal pattern (Fig 1).

**Fig 2:**
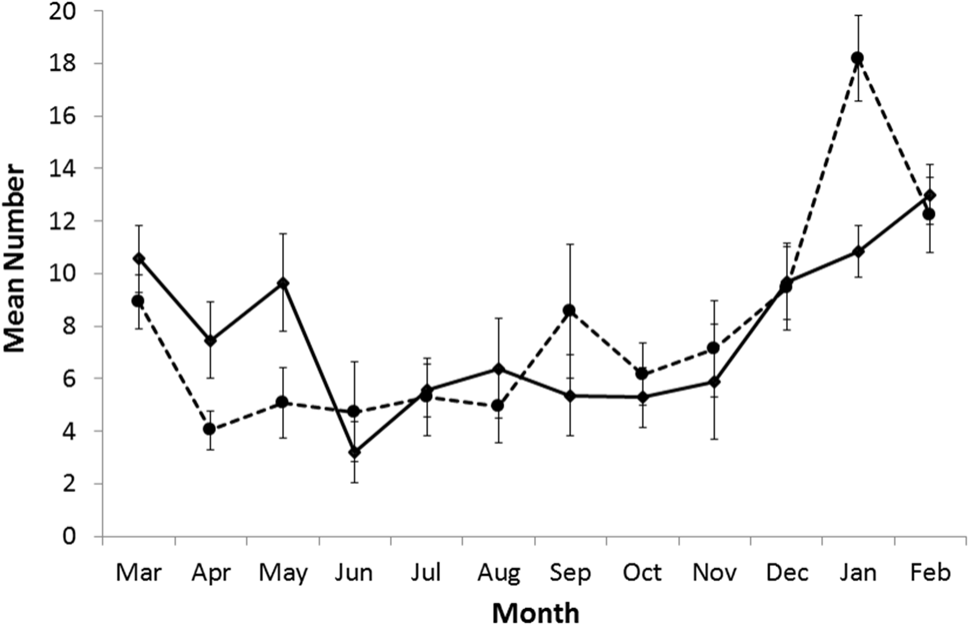
Mean number of follicles for females in each month. Mean number of secondary (broken line) and tertiary (solid line) follicles for females by month. Error bars are standard error of the mean.

The development and maturing of tertiary follicles was observed throughout the year and they were present at a high frequency in all age classes. Females with secondary and tertiary (pre-ovulation) follicles were observed in each month (Fig 2). The monthly mean numbers of secondary follicles was 9.1 (range 0–47) and for tertiary follicles 8.8 (range 0–37). For both stages of follicular development the numbers showed wide month to month variation. There was a marked peak in secondary follicles in January that coincided with the peak in parturitions. The peak in tertiary follicles extended from December to May (Fig 2). The highest number of tertiary follicles was seen in February–March, the period immediately following parturition and the beginning of the breeding year. It was clear that follicular development was independent of breeding status: the average number of tertiary follicles in sows carrying blastocysts was 7.9, in gravid sows it was 13.0, in post parturient sows it was 10.4 and for non-pregnant sows during February–March it was 11.0 (Chi squared test: χ^2^ = 1.25, df = 3, p-value = 0.7404).

The age of the sows also did not affect the proportion of individuals with tertiary follicles, as tertiary follicles were seen in 100% of the juveniles, 98% of yearlings, 87% of adults and 94% of aged adults. All juveniles showed high numbers of secondary follicles (mean 25, range 22–29) but fewer tertiary follicles (mean 11.8, range 5–23). The four juveniles sampled in January—February had a greater number of secondary follicles (22, 23, 24 and 29, mean 24.5) than the other females (mean 13.9) at that time (Wilcoxon rank sum test with continuity correction W = 252, N = 4, 74, p = 0.02). There was a similar difference for the June-July period (Wilcoxon rank sum test with continuity correction W = 58, N = 2, 29, p = 0.02) with juveniles carrying more secondary follicles (25, 28, mean 26.5) than other females (mean 3.6). No significant difference was found in the tertiary follicle numbers between age classes, and no effect of month was found in the secondary or tertiary follicle numbers.

**Fig 3:**
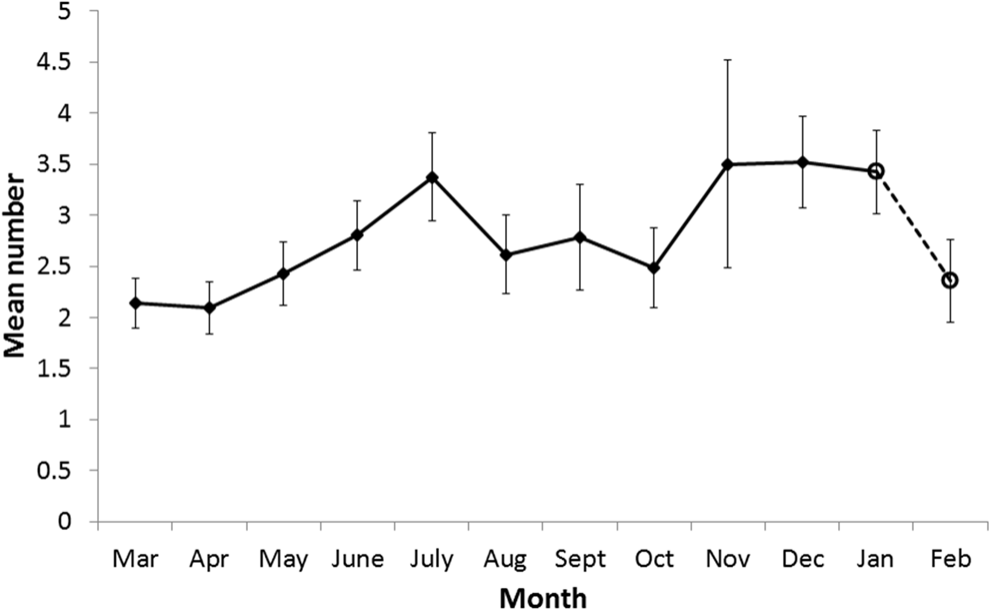
Mean number of corpora lutea in each month. The mean number of corpora lutea found in each month in females carrying blastocysts (diamond marker and solid line) or foetuses (open circular markers and broken line). Error bars depict standard error of the mean.

Females with corpora lutea (CL) were observed throughout the year. The mean number of CL carried by females increased from a low (mean 2.1) in March and April to a peak in November–December (mean 3.5) (Fig 3) (Wilcoxon rank sum test with continuity correction W = 633, N = 58, 31, p = 0.017).

**Fig 4:**
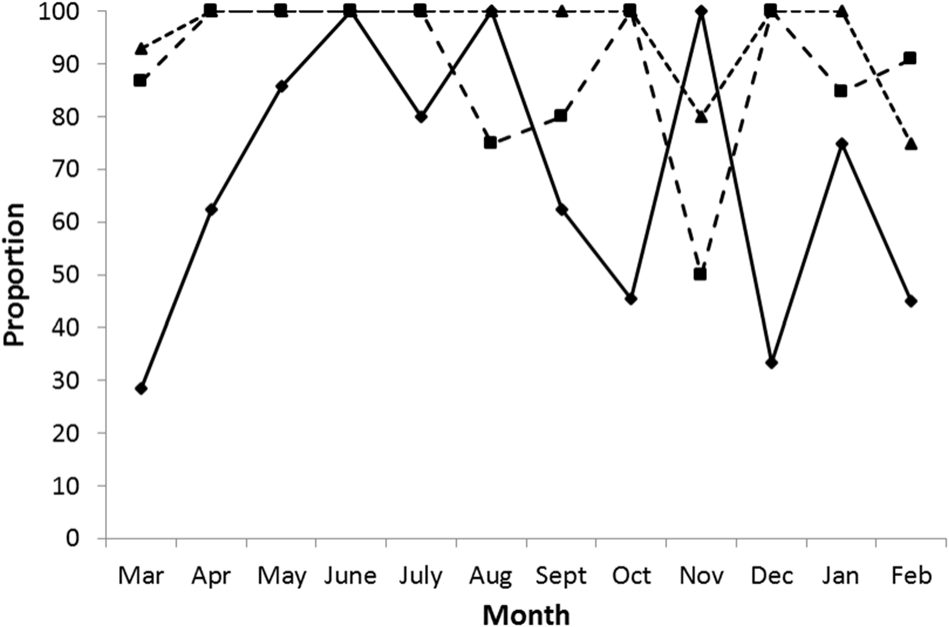
Effect of age on corpora lutea occurrence in each month. The percentage of each age class carrying corpora lutea by month: yearlings—diamond marker, adults—square marker and aged adults—triangular marker.

Although the numbers carried did not differ with the female’s age, there was an effect of age on the proportion of females with CL (Fig 4). Using a binomial regression, we identified that badger age, season and badger body condition were all important predictors of whether an animal would be carrying corpora lutea (Table 2). The aged females were more likely to have CL than the adults or yearlings, and CL were more commonly found in summer than the other seasons. However the strongest influence on whether a CL was present was the body condition of the female (Table 2).

**Table 2:** Results from a binomial regression on the presence of corpora lutea in female badgers between March and December for different age classes (age), seasons and body conditions (quality–a value derived by dividing bodyweight by body length). Age and Season were treated as categorical variables in the model, whereas quality was treated as a continuous variable. Body condition values were normalised (z.Condition) prior to analysis. Asterisks indicate significance level.

	estimate	Std. Error	Z value	P value
intercept	0.9104	0.4449	2.046	0.041 *
Season Spring	0.2005	0.498	0.403	0.69
Season Summer	1.722	0.6605	2.607	0.009 **
Season Winter	-0.6706	0.457	-1.467	0.14
est. age Aged	1.0688	0.4783	2.235	0.025 *
est. age Yearling	-0.4972	0.3324	-1.496	0.135
z.Condition	1.637	0.418	3.913	0.00009 ***

Asterisks denote significance where

* = P<0.05

** = P<0.01

*** = P<0.001.

The seasonal variation was greatest in yearlings, with little monthly variation in the proportion of adults and aged adults carrying CL (Fig 4). In the first 6 months of the breeding year the proportion of yearlings with CL rose from 29% in March to 100% in June and August, and then declined to 33% in December. In adult and aged females, except in November, the proportion carrying CL remained in the vicinity of 100% all year. The wide fluctuations seen in November (Fig 4) could possibly be due to age misclassification between yearlings and adults since yearlings at this point would be nearly two years old and so not easily differentiated from adults. The sample size for this month was also relatively low (Table 1). All females that were either gravid or postpartum had CL. There were five females with blastocysts (either one or two blastocysts) in which no CL were detected. For those with blastocysts and CL the mean was 3.8 CL per female but for gravid females the mean was 4.9, and in postpartum females 4.7. In the 122 females with no detected blastocysts, there was a mean of 3.7 CL.

### Blastocysts

**Fig 5:**
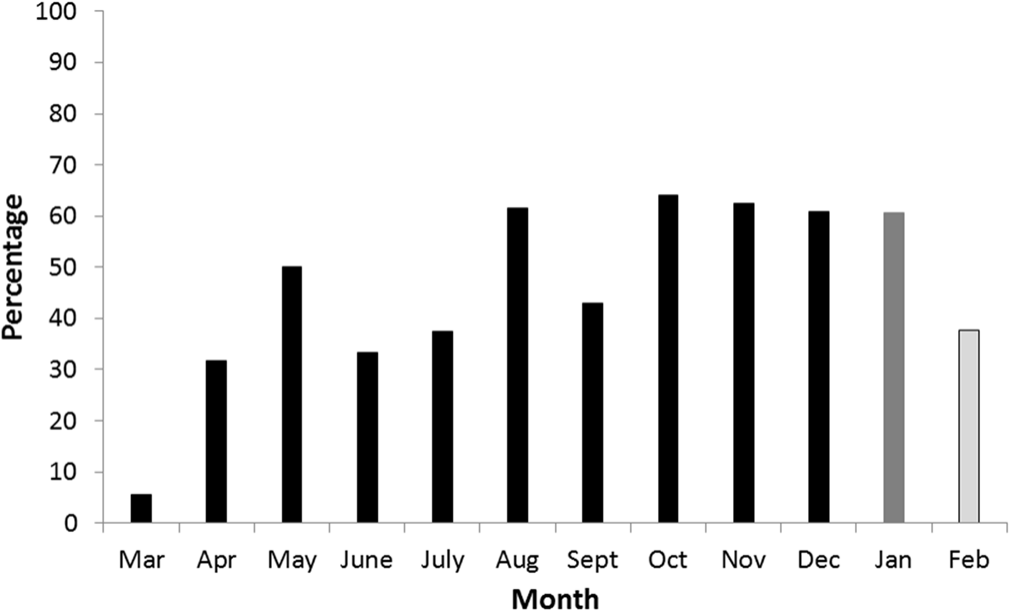
Proportion of females with blastocysts in each month. The proportion of females, by month, carrying blastocysts (back bars, March to December), foetuses (dark grey bar, January) or that were postpartum (light grey bar: February).

**Fig 6:**
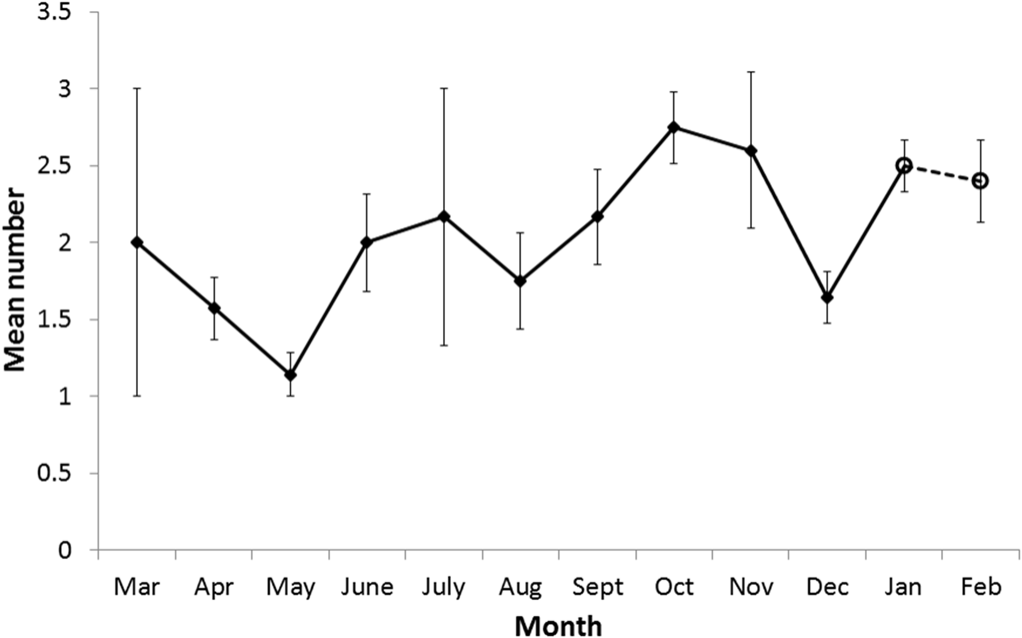
Blastocyst and foetus mean numbers each month. Mean number of blastocysts (diamond: March to December) or foetuses (open circle: January and February) carried by pregnant females by month. Error bars depict standard error of the mean.

Blastocysts were first observed in March and the last were observed in December (Fig 5). Blastocysts were recovered from 5.7% of females in March. This proportion increased to 50% in May and reached a plateau of 62.5 to 66.7% between October and December. The mean annual number of blastocysts per pregnant female was 2.14 and the monthly mean fluctuated around this number (Fig 6) with the lowest number in May (1.14) and the highest in October (mean 2.75). There was a significant increase in blastocyst numbers through the year, from May to October (Pearson's product-moment correlation t = 3.25, df = 45, p-value = 0.002). In December, the period during which implantation occurred, the mean number of blastocysts reduced to1.6, which was not significantly higher than the number of blastocysts per female recorded for March (Wilcoxon rank sum test with continuity correction W = 15, N = 2, 13, p = 0.78). No female classified as postpartum was found to be carrying blastocysts.

Mean blastocyst numbers did not differ by age class: yearlings had a mean of 1.79 blastocysts per individual; adults had a mean of 1.93 and aged adults had a mean of 2.3. A two-way ANOVA of blastocyst number for the period May to October, showed a highly significant increase in blastocyst numbers with time (P = 0.003), but no effect of age on blastocyst numbers (P = 0.485) and no interaction between age and month (P = 0.560) (Table 3).

**Table 3:** ANOVA test results for the number of blastocysts carried by badgers between May and October (month) for different age classes (age) and the interaction between age and month (interaction).

	Df	Sum Sq	Mean Sq	F value	Pr(>F)
month	1	11.02	11.017	10.246	0.0027
age	2	1.59	0.793	0.738	0.485
interaction	2	1.27	0.633	0.589	0.560
Residuals	41	44.09	1.075		

**Fig 7:**
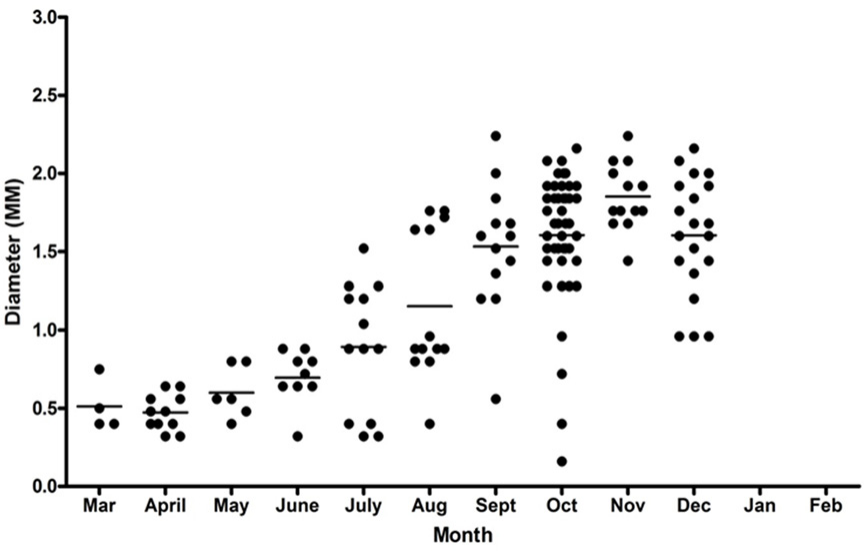
Diameters of blastocysts each month. Distribution of blastocyst diameters by month. The horizontal line indicates the mean blastocyst diameter for that month.

The diameter of blastocysts ranged from 0.16 to 2.24 mm with the mean monthly diameter increasing progressively throughout the year, (Pearson's product-moment correlation: March–Dec t = 5.77, df = 148, p <0.001) (Fig 7). Blastocysts in the period March to June were all relatively small, with diameters in the range 0.3–0.9mm. The range of blastocyst sizes increased through the year with both small and large ones present in July to October (Fig 7). The size range of blastocysts, both in individual females and at any time of the year were diverse.

Females with multiple blastocysts sometimes carried more than one blastocyst of the same size. Of the 24 females which carried two blastocysts (Table 4) in 18 they differed in size, and in six they were of equal diameter. Of the 18 females with three blastocysts, in 13 they all differed in size and five had two blastocysts of equal diameter. In the one female with four blastocysts, three were of equal diameter. One female had six blastocysts, two large and of equal diameter and two small and of equal diameter. The diversity of the blastocyst diameters was also evident when the range of sizes carried by an individual female was considered. In 37 of the 47 females that had multiple blastocysts, the difference in diameter of the largest and smallest was less than 20%. In the remaining 10 females, in four there were very marked blastocyst size differences. In one female with two blastocysts the difference was 70%, in one with three it was 74%, in one with four it was 80%. One female had six blastocysts differing in size by 73% and of four distinct sizes: two at 1.2mm, one at 1.04mm, one at 0.84 mm and two at 0.32mm. This last female was sampled in July, and it is interesting that her smallest blastocysts were within the size range seen in March when blastocysts were all newly formed.

**Table 4:** Number of females carrying a given number of blastocysts.

No. Blastocysts	1	2	3	4	5	6
No. Females	29	24	18	4	0	1

The mean diameter of blastocysts was 1.17mm in yearlings, 1.21mm in adults and 1.50mm in aged adults A two-way ANOVA across months and ages showed that although the size of blastocysts increased significantly with time (P< 0.001), there was no effect of the age of the animal on the size of blastocysts carried (P = 0.795), and no interaction between age and month (P = 0.962) (Table 5).

**Table 5:** Results from a 2-way ANOVA on the size of blastocysts carried by badgers between March and December (month) for different age classes (age) and the interaction between age and month (interaction).

	Df	Sum Sq	Mean Sq	F value	Pr(>F)
month	1	27.671	27.671	189.246	> 0.001
age	2	0.067	0.034	0.230	0.795
interaction	2	0.011	0.006	0.038	0.962
Residuals	144	21.055	0.146		

### Progesterone concentrations

**Fig 8:**
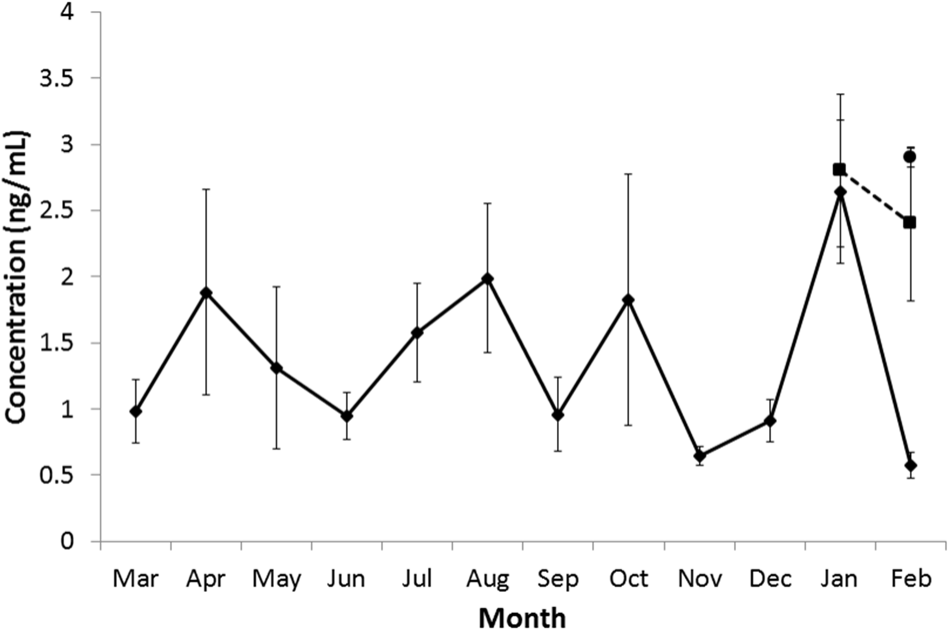
Mean circulating progesterone concentrations in each month. The mean concentration of circulating progesterone (ng/mL) by month. Error bars depict standard error of the mean. Diamond markers–mean for all females, square markers–mean for gravid females, circular marker–mean for postpartum females.

Mean monthly progesterone concentrations increased in an inconsistent fashion over the breeding year to a maximum in January (Fig 8). In January the progesterone concentrations in gravid females were not different from the population mean, but in February the gravid females maintained high concentrations of progesterone, while in the postpartum females and the rest of the population concentrations of progesterone became very low (Fig 8). A comparison between the progesterone concentrations found in gravid and non-gravid females in January found no significant differences (Wilcoxon rank sum test W = 95, N = 16, 8, p-value = 0.061) but in February the gravid females had significantly higher progesterone concentrations than non-gravid or postpartum females (Kruskal-Wallis rank sum test Kruskal-Wallis chi-squared = 12.0428, df = 2, p-value = 0.0024; post hoc Wilcoxon rank sum tests: gravid vs non-gravid W = 125, N = 7, 20, p-value = 0.00127; gravid vs post-partum W = 39, N = 7, 6, p-value = 0.0082; non-gravid vs post-partum W = 34, N = 20, 6, p-value = 0.1229).

Progesterone concentrations were not correlated with numbers of corpora lutea for either gravid or non-gravid females (Pearson's product-moment correlations, gravid females t = 1.19, df = 21, p-value = 0.248, non-gravid females t = -0.73, df = 174, p-value = 0.466).

### Gravid Females

The first gravid female was captured on 17^th^ January and the last on 21^st^ February, and it was only during this period that postpartum females were also observed. In January and February 78 females were examined of which 30 were gravid and 6 were postpartum. The 30 gravid females carried 74 foetuses (mean 2.47, range 1–4). During the period when gravid females were present in the population 32 yearlings were examined of which 13 were gravid (mean 2.46 foetuses) and one was postpartum, 29 adults were examined of which 11 were gravid (mean 2.72 foetuses) and three were postpartum, and of 13 aged adults examined six were gravid (mean 2.0 foetuses) and two were postpartum. There was no difference in the number of foetuses across age classes (Wilcoxon rank sum tests with continuity correction: yearling vs adult W = 59, N = 13, 11, p-value = 0.43, yearling vs aged W = 50, N = 13, 6, p-value = 0.33, adult vs aged W = 17, N = 11, 6, p-value = 0.076). The results above give overall implantation success rates for yearlings of 43.8%, adults of 48.3% and aged adults of 61.5%, showing no significant change in implantation success with age (Chi-Squared test; χ^2^ = 3.31, df = 2, p-value = 0.19).

In October, (when the females were carrying blastocysts but not foetuses), 16 pregnant females carried 44 blastocysts (mean of 2.75 per pregnant sow), in November five pregnant females carried 13 blastocysts (mean of 2.6) and in December 14 pregnant females carried 23 blastocysts (mean of 1.64). Thus the number of blastocysts in the three months preceding implantation correlated well with the number of foetuses observed.

## Discussion

Our sample of female badgers was drawn from a wide geographic range and is representative of the population in the Republic of Ireland. Throughout the year there was a high level of reproductive activity in the female badger population. Females with developing secondary and tertiary follicles were seen throughout the year, with 86% carrying tertiary follicles. The badgers are therefore likely to have been physiologically receptive to mating throughout the year [4]. There was an extended peak in the number of tertiary follicles between January and May but there was no autumn–spring peak as described by Cresswell et al. [19]. When the data for yearlings was excluded, there was a constant high proportion of females with corpora lutea across the year. The mean number of corpora lutea rose from a low in April to a peak extending from November to January. The mean monthly number of blastocysts fluctuated around the annual mean of 2.0 (range 1–6) with an increase in the mean number of blastocysts from a minimum in May (1.14) at the beginning of the breeding year to a maximum in October (2.75). It was not until late summer to mid-autumn that the majority of females were found to be pregnant. The proportion that were pregnant increased to around 60% by August and in January 60% were gravid. Gravid females were only seen in winter, in a six week period in January and February, and those immediately postpartum were only seen in February. The mean number of foetuses carried by gravid females was 2.5 (range 1–4), similar to the number of blastocysts seen in the months immediately preceding implantation. In a previous study of Irish badgers, in East Offaly, the mean number of foetuses per gravid female was similar, at 2.9 (range 1–4) [22] although Whelan and Hayden’s [22] study did not distinguish adult and aged badgers.

Our findings in Irish badgers are, in general, consistent with those of previous studies in Ireland [22], Sweden [18], and Britain [19, 20]. If we use the pattern of blastocyst accumulation as an indicator of mating, we saw, as reported in Britain, that badgers may mate during any month of the year. However, in our study we did not see a peak in matings occurring in late winter to summer which was observed in England by Cresswell et al. [19]. The peak in matings observed in our study was later, not occurring until late summer and early autumn, with no difference between yearlings and adults. Our findings also contrast with those of Ahnlund [18] in Sweden, where matings were reported as being confined to a concise period immediately postpartum, starting in March and rising to 100% fertilisation rates in adults by June, and somewhat slower rise to a peak in December for yearlings.

In our study, progesterone concentrations varied widely during the year both between months and between individuals. It reached a peak in all females in January. It remained high in gravid and postpartum females, but dropped to very low levels in non-pregnant females in February. Although we observed a greater number of corpora lutea at the time of the progesterone peak in January than at other times, there was no correlation between the number of corpora lutea and the progesterone concentration. It is likely that the production of progesterone would decrease with the aging of the corpora luteum, [45] which may necessitate females to continue ovulating throughout the period of delayed implantation to maintain effective progesterone levels.

We found that only 66% of the female population conceived and a similar proportion successfully implanted. The mean number of foetuses in January and February (2.5) was equal to the number of blastocysts recorded in October and November. The conception rate found in our study was lower than that reported for any other study of European badgers. In Irish badgers studied by Whelan and Hayden [22] the conception rate was 80–90%; while a conception rate of 80% was reported for badgers in Great Britain by Page et al. [20]; in Sweden, the conception rate was reported as 100% for adult females but only 70% for yearlings [18]. Despite our study badgers having a lower conception rate, they had a very high implantation success, which was not different from the conception rate. Whelan and Hayden [22] concluded that implantation losses occurred because there was a decrease in the pregnancy rate before and after implantation (80–90% to 65–70%), especially in yearlings with a decrease from 65% to 35%. Even greater losses are reported in badgers in Great Britain, with only 44% of badgers implanting as reported by Cresswell et al. [19], while Page et al. [20] reported that 80% of pregnant females failed to implant and Carpenter et al [46] reported only a 30% reproductive success rate. Even where females did implant there were losses as Cresswell et al [19] and Page et al. [20] found 3.2–3.4 blastocysts per pregnant female in November and December but only 2.9 foetuses per gravid female. Both Whelan and Hayden [22] and Page et al. [20] reported mean litter size of 2.9, which is comparable to our mean litter size of 2.5. Thus despite lower conception rates, the subsequent breeding success of Irish badgers was higher than that reported in Great Britain.

We did not detect any foetal deaths as was reported in English badgers by Page et al. [20] and Roper [30] who reported about 20–30% of pregnant females lost all their foetuses while an additional 10% suffered partial losses. Foetal deaths have been seen in a study of Irish badgers in which 26 gravid females were examined post mortem and in each of two litters of three cubs, one cub had died and was decomposing (Corner, pers.obs.). However the present study indicated a greater survival rate of foetuses than expected from studies of English badgers.

Many lines of evidence from our study demonstrate that during the period leading up to implantation the blastocyst population was dynamic, with a continuous turnover of blastocysts. We found a constant presence of tertiary follicles, histological evidence of oestrus, and an increase in corpora lutea indicating ovulation. In addition, the number of corpora lutea exceeded the number of blastocysts and the number of corpora lutea bore little relationship to the number of blastocysts. The mean number of blastocysts remained around 2.0 until mid-year, when it rose to over 2.6 in October and November. Females continued to cycle throughout the year, producing new blastocysts, and as the number present was constant through most of the year, blastocysts must have been lost at approximately the same rate as they were produced. Later in the year the rate of production of new blastocysts must have marginally exceeded the rate of loss, as the numbers observed rose slightly. No ovulations were observed after implantation. Thus our results agree with those of Neal and Cheeseman [47] that female badgers continue to cycle throughout the year, even when carrying blastocysts but not when gravid. However our results contrast with those of Ahnlund [18] who concluded that the replacement and additions to the number of blastocysts was a rare event in his population.

Further evidence of the dynamic nature of the blastocyst population is the change in blastocyst diameter with time. In the badger, embryonic diapause is not associated with suspended growth of the blastocyst. Embryos remain metabolically active and continue to grow [2]. We observed that the average diameter of unimplanted blastocysts continued to increase as the year progressed. Others have made similar observations of badger blastocysts, in Ireland [22], in Sweden [18] and in Great Britain [19, 20]. We observed, again as others before us, that even though blastocysts continued to increase in diameter, there was a continued presence of small blastocysts in some females, including small blastocysts among larger ones in individual females. Cresswell et al. [19] and Ahnlund [18] considered these latter small blastocysts as being associated with yearlings coming into oestrus and Page et al. [20] considered them a second generation of blastocysts. We observed no association with age of the females, and first observed distinctly smaller blastocysts in July in females with multiple blastocysts. From July onwards, both within individuals and across the badger population, small blastocysts (<1.0mm diameter) were present among larger ones: the smaller size was typical of those seen in the early months of the year. Superfetation is the production of new embryos after the female is already pregnant [3]. In our sample, 47 (62%) of the 76 females with blastocysts had more than one present at a time; ten (21%) of these females were carrying blastocysts with size differences of more than 20%. Thus our results represent multiple examples of superfetation in the females in this population.

The yearling badgers in the study had very similar rates of breeding success to those found for adult badgers and there was no significant difference from the success rate for aged females. Thus there was no evidence of breeding suppression by adult females of yearlings in contrast to the findings of Carpenter et al. [46] who found over a 14 year period in a high density English population a deficit in the breeding performance of 2nd year females.

Both embryonic diapause and superfetation have the potential to increase female reproductive success. Embryonic diapause allows the female to delay implantation so that her cubs are born at a time of year which coincides with high food availability. In addition it could allow the female to mate all year round, increasing her chances of mating with a variety of males [4]. Carpenter et al. [46] combined field data with genetic data (16 microsatellite loci), in studying the mating behaviour across 14 years in a high-density population. They were able to assign parentage to 47% of the cubs (80% confidence) and roughly half of assigned paternity was attributed to extra-group males. Superfetation could provide females with increased assurance of reproductive success in the face of blastocyst losses during embryonic diapause. In addition, superfetation may be an evolutionary consequence of the need to maintain a suitable uterine environment for blastocyst survival. The ovulations which provide these blastocysts also produce additional corpora lutea to replace involuting corpora lutea, or simply to increase their number. These may be needed to maintain an adequate progesterone concentration required for maintenance of the uterine environment [29].

Another reproductive advantage of superfetation allied with embryonic diapause, may be hybrid vigour in the cubs if the second male is less closely related to the female than earlier mates. Such out-breeding, and associated hybrid vigour, have been shown to increase the genetic difference between the foetus and the mother and that this increases the chance of a successful completion of the pregnancy [4, 48, 49].

Finally, the ability of females to mate throughout the year, and for some of those matings to lead to successful fertilisations, would mean that the males could not predict which of their matings were likely to father cubs. This would effectively prevent males from practising infanticide, as the paternity of any given cub would be uncertain [4], and reduce any benefit of mate guarding since they would have to maintain this behaviour all year. Thus superfetation may be an adaptation which allows females to practice effective polyandry without males being able to counter this strategy.

This study provides a clear demonstration of the presence of superfetation in badgers in Ireland, and provides strong evidence that the breeding rate in Irish badgers is limited by failure to conceive, rather than failure at any other stages of the breeding cycle. There were few effects of age on breeding success in this population, suggesting no breeding suppression by adult females. This study highlights a number of significant differences between the reproductive biology of female Irish badgers from those of Great Britain and Swedish populations.
